# Communication and resolution programs expose hard-to-hear truths

**DOI:** 10.3389/frhs.2024.1523363

**Published:** 2025-03-04

**Authors:** Gerald B. Hickson, Richard C. Boothman, Alice M. Krumm, Ronald Wyatt

**Affiliations:** ^1^ Center for Patient and Professional Advocacy, Vanderbilt University Medical Center, Nashville, TN, United States; ^2^ Boothman Consulting Group, LLC, University of Michigan Medical School, Ann Arbor, MI, United States; ^3^ Independent Researcher, Orange Beach, AL, United States

**Keywords:** communication and resolution program, professionalism, team member wellbeing, disclosure, medical error

## Abstract

Communication and Resolution Programs' (CRP) favorable impact on professional liability claims continues to draw attention, but because they are deliberately aligned to advance the health system's mission rather than amelioration of litigation exposure, CRPs stand a better chance of delivering durable healthcare improvements than traditional responses to patient harm. CRP adherents employ focused investigations overseen by their own patient safety leader in order to engage patients with a principled response following unintended clinical outcomes. Focused on safety and unencumbered by litigation delays, CRP investigations are more apt than traditional responses to lay bare patient safety risks including professionalism challenges. Leaders, however, must be prepared to embrace and address hard-to-hear truths about dysfunctional systems or disruptive humans that threaten outcomes of care or clinical staff wellbeing.

## Introduction

Communication and resolution programs (CRPs) are a principled, comprehensive, and systematic approach for responding to unintended patient harm. Their avowed goal is to promote rapid clinical improvement while treating the injured patient and family consistently with the organization's clinical mission: honestly and empathically. CRPs are founded on three organizational commitments: Patients are always entitled to honesty and transparency, but when inappropriate care causes harm, patients receive an apology and an offer of fair compensation. When an unplanned clinical outcome occurs despite reasonable care, the patient still receives an honest and compassionate explanation. And, most importantly, the health system leverages lessons learned to quickly improve performance (1). The first two, aligned with clinical expectations, nurture accountability. The third principle leverages the speed and honesty of the first two to the benefit of other patients, healthcare professionals, and the safety culture. These principles require honest assessments of clinical performance (not litigation-based assessments of defensibility), a significant shift from traditional dependence on assessments of courtroom chances. Freed from litigation's delays and gamesmanship, CRPs conspicuously prioritize safety, patient centricity, and support for medical team members (2, 3). A word of caution to leaders: to take full advantage of their CRP's promise, be prepared to embrace and address hard-to-hear truths that threaten outcomes and your organization's pursuit of a culture of safety and respect.

## A hypothetical case

Seventy-five-year-old Mr. Patient presented with abdominal pain, distention, nausea, and vomiting. The findings suggested a complete bowel obstruction secondary to a right colon mass. Mr. Patient was scheduled for an open right hemicolectomy as an emergent add-on case for Dr. Surgeon. Anesthesia resident, Dr. Learner, prepared for general anesthesia.

On postoperative day three, a CT scan per protocol to assess the integrity of the anastomosis revealed a retained foreign body (RFB). A surgical sponge was subsequently removed. The RFB was reported through the hospital's safety reporting system.

Per this hospital's CRP process, system leadership was notified. The Safety/Risk team initiated a case review with the operating room (OR) team member interviews. The OR team was aware of the count policy, but explained that the case was an add-on, the team was busy, and the count was erroneously documented as correct. The findings were shared with leadership. Deemed an avoidable event with consequences, plans were made to speak with the patient.

A Leadership Team (Chief of Staff, Perioperative Nursing Lead, Risk Manager, and Dr. Surgeon) was scheduled to meet with Mr. Patient to review the case findings, apologize, and introduce the subject of compensation. Just before the meeting, Dr. Surgeon reported being called away to an emergency. The other leaders chose to meet Mr. Patient at the bedside.

The Leadership Team explained the case findings. Anticipating Mr. Patient's concerns, they described a plan, including re-evaluation of the count policy, calling out count status after the second count, re-education of nursing on the new policy, and conducting count audits on all shifts for six months (4–7). Mr. Patient received the apology and was informed the mistake and harm deserved compensation. He appreciated the apology and the plans to prevent similar mistakes from impacting others, but surprised them with, “That’s fine, and it’s exactly what Dr. Surgeon said you would say when we talked this morning … What I, and at least one important member of your surgical staff, Dr. Surgeon, wants to know however is, ‘What are you going to do about the incompetence of your second-string nursing team who can’t count?’ Dr. Surgeon told me it is hard to focus on surgery while having to keep one eye on rookie nurses. He said it was chaos in that OR!”

Surprised, the leaders excused themselves and promised to get back to Mr. Patient. The group immediately met to consider what they heard, wondering how the CRP investigation did not capture the surgeon's apparent version. The Leadership Team immediately interviewed Dr. Surgeon who summarized his assessment: “The nurses were inexperienced, can't count or know when to ask me to order a film.”

The leaders directed a closer focus on team performance. They dispatched a patient relations representative to Mr. Patient to document the story he had previously shared, and any additional concerns he might have (8). However, deeper inquiries failed to support Dr. Surgeon's assertions. The OR team, an RN Circulator and a Surgical Tech, were neither rookies nor incompetent. Both worked evening shifts and had relieved the OR team after Dr. Surgeon's last scheduled case. Both had more than 5 years of experience. Their performance evaluations exceeded expectations. RN Circulator recalled another recent event reported anonymously, “I asked Dr. Surgeon to pause before closing. We had no tech to support the count. He ignored me and started closing.”

The picture that emerged differed from Dr. Surgeon's assertions to both Mr. Patient and the Leadership Team and raised concerns about the surgeon's behavior. “Dr. Surgeon entered the OR, pushed the door so hard it slammed against the wall and announced, I need a patient, NOW!” one team member reported. Another team member shared, “Dr. Surgeon asked Dr. Sleep (attending anesthesiologist) if he could talk to him briefly.” We all overheard Dr. Surgeon insist, ‘Dr. Sleep, YOU get this patient to sleep. It's bad enough I've got to deal with second-string nurses, let alone the rookie resident, Dr. Learner.’ Dr. Sleep reportedly dismissed Dr. Learner, ‘It's OK, I'll take over.’

The Leadership Team learned that upon re-entering the OR, Dr. Surgeon declared condescendingly, “Now, listen carefully to our time-out so I don't get reported again. I'm Dr. Surgeon. We're dealing with an obstruction. Questions?” No one, including Dr. Sleep, replied. Throughout the case Dr. Surgeon made sarcastic remarks about how slow everyone was moving. The OR team recalled that as he began to close, Dr. Surgeon interrupted their sponge counts, instructing the RN Circulator to answer a page from Dr. Ed in the emergency room, at one point brusquely instructing the nurse, “Hold the phone to my ear so I can close while I finish this conversation!” and at one point, yelling at RN Circulator, “Stop moving so I can talk on this #@*% phone!” When RN Circulator asked after the call, “Could we have a minute to repeat the count?” Dr. Surgeon stared and declared, “DONE! Did everyone hear? DONE!” Counts were marked as correct, and Mr. Patient was transported to recovery.

The OR team characterized the room as more “toxic” than “chaotic.” The counts were clearly incorrect, no film was taken, a sponge remained in Mr. Patient, and his recovery was complicated by an avoidable error. The RN Circulator responded to a Leadership Team member, “I thought my counts were correct, but they weren't. It was not a complex case, but I don't know how anyone could count in that environment. I just wanted out of the room.” The Leadership Team regrouped to consider the situation, messier than first reported. Reassured that the patient would be treated fairly, they turned to three hard-to-hear concerns that needed to be addressed:

• Does Dr. Surgeon represent a threat to safety, staff wellbeing, and to his own wellbeing?
• How did we get here? How could our health system tolerate or even enable the behaviors described? As leaders, have we contributed?
• We thought we had a reliable CRP, are we wrong?

## A priority: safety

CRP operational elements specifically prioritize safety first, not litigation exposure, and tackle challenges identified whether related to systems that need attention or humans who may be struggling and need help (2, 9–11). Not only did leaders discover a toxic work environment, but other stories suggested the behaviors described an established pattern. An OR team member commented, “I've reported Dr. Surgeon before. Nothing happens. Instead, we get his stares or worse.” Leadership summarized their immediate priorities in a couple of questions that need to be addressed:

• Is Dr. Surgeon well and safe to practice?
• Why was the unprofessional behavior experienced by the surgical team not reported and addressed by leadership?

The Leadership Team called an urgent meeting with Dr. Surgeon's department chair, Dr. Leader, and the General Counsel. Dr. Leader acknowledged that Dr. Surgeon could be challenging but kept insisting he is “a skilled, productive surgeon and important to our practice”. Agreement was finally reached to initiate a comprehensive review of Dr. Surgeon's practice and behavior including a mental and physical health evaluation.

A small subset (4%–6%) of physicians and nurses model disrespectful behaviors in practice (12–14). Their disrespect for patients, coworkers, and established safety practices threatens outcomes of care (12, 15–19). Medical team members subjected to disrespect are less vigilant, and less likely to communicate and ask for help (20, 21).

Patients of physicians who model disrespect are 20%–30% more likely to experience avoidable surgical and medical complications, and death (15–18). Compared with same specialty peers, physicians who model unprofessional behavior create needless malpractice claims and costs (12, 19). Furthermore, “high risk” today means “high risk” tomorrow (22). Failing to confront disrespectful team members leads to substantial but preventable economic and non-economic costs (23–25).

**Figure 1:**
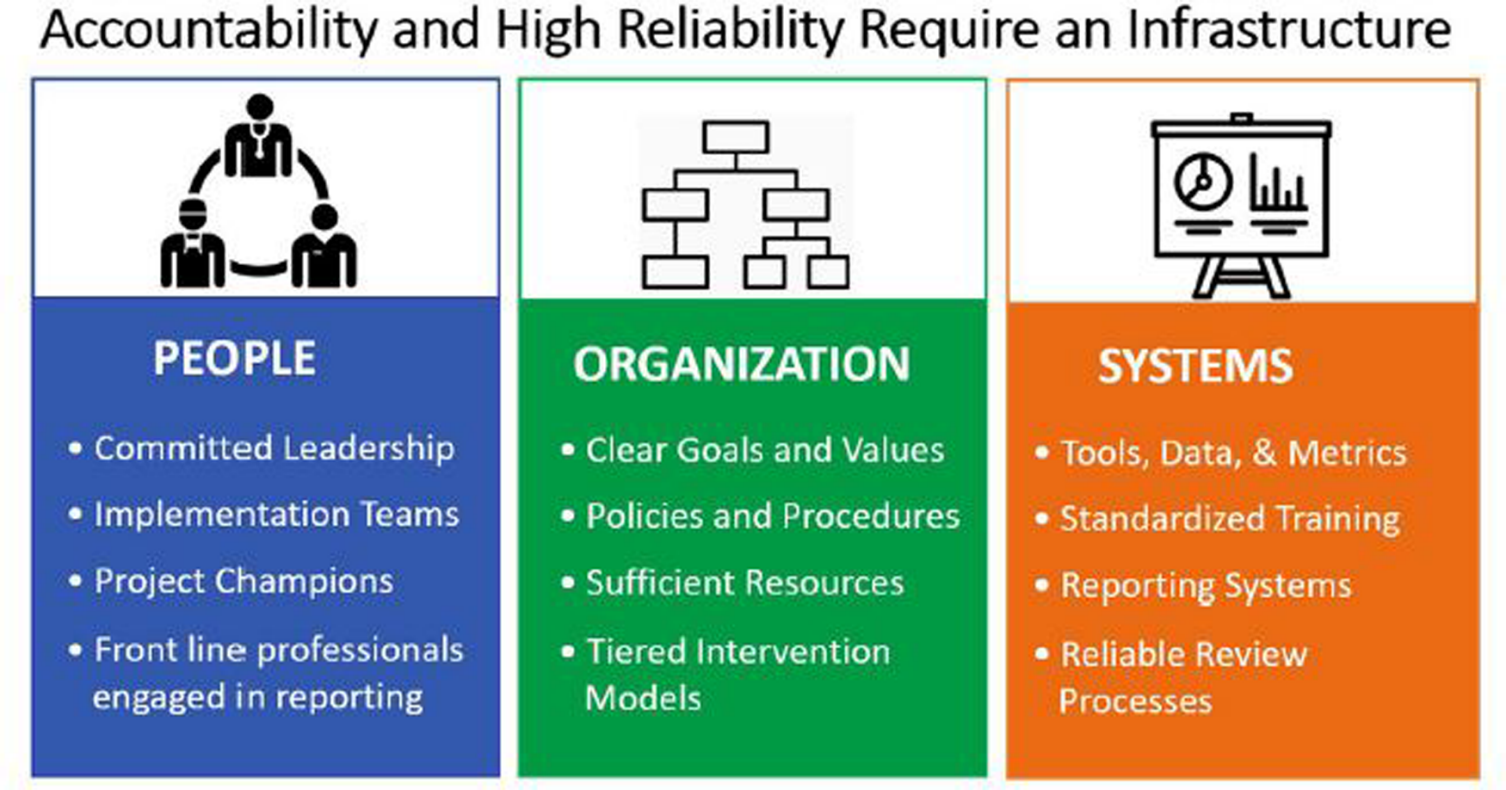
Structural elements for promoting accountability and high reliability. People, organization, and systems are the building blocks for supporting professional accountability From Joint Commission Resources.

When a CRP investigation identifies patterns of disrespect, intervention is key, but also required is a plan supported by a well-defined infrastructure (Figure 1) that links the right people, organization, and system support to identify and address every system's Dr. Surgeon in a fair, effective, and timely manner (26–28). Leaders who model professionalism and commit to holding all team members accountable to consistent expectations of behavior and performance, regardless of rank or perceived value, are the right people (29, 30). These are real leaders, not apologists or protectors who might insist, “But Dr. Surgeon is skilled and VERY productive.”

**Figure 2:**
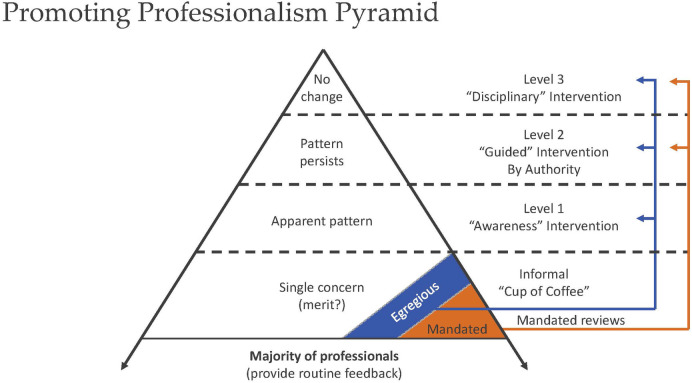
A tiered intervention model to guide sharing stories and data to promote professional accountability. Adapted with permission from “The disruptive behavior pyramid for identifying, assessing, and dealing with unprofessional behavior” by Gerald Hickson, James Pichert, Lynn Webb, Steven Gabbe, licensed under CC-BY-ND.

Other infrastructure elements include synchronizing policies and processes with a system's core values directing a tiered approach (Figure 2) for sharing unprofessional/disrespectful behaviors reported by coworkers or patients beginning with peer delivered “Coffee” for single non-egregious events progressing to leader-directed corrective action plans for the small percent who are unable or unwilling to self-regulate (26, 27, 30, 31). Corrective action is intended to connect struggling professionals with appropriate resources, including coaching, physical and mental health screening, and treatment as indicated (30, 31). Persistent unprofessional behavior is associated with mental illness, substance abuse, significant life stressors, early cognitive impairment, and burnout (32–34). Although a regular observer of Dr. Surgeon's behavior, Dr. Sleep remained silent in the face of behaviors that called for respectful, in-the-moment feedback or submission of a safety report.

The Leadership Team met with Dr. Anesthesia Leader to seek answers to two questions:

• Who will talk with Dr. Sleep about what appears to have been missed opportunities to support safety and fellow professionals and afford a suitable learning environment for Dr. Learner?
• And who will debrief Dr. Learner about the unhealthy experience and coaching, should Dr. Learner encounter a similar circumstance again? (35, 36)

As the leaders departed, Perioperative Nurse Lead shared that meetings had occurred with the nursing professionals involved to debrief and make them aware of available wellness services and a commitment by leadership to address any form of retaliation directed at professionals who report safety concerns (30).

## Team member silence?

Medical team members often choose not to speak up about the kind of safety challenges posed by Dr. Surgeon—why? (36–39). There was no trust. As a team member shared, “I've reported before. Nothing happens. I've quit reporting.” Willingness to report safety concerns is influenced by individual, team, and organizational factors, but mostly trust that the reporter will not be humiliated, suffer retaliation, and that good will come of their courage to speak up (38–40).

Leaders must actively promote speaking-up. Individually, they can model a desire to listen and a determination to respond constructively affirming everyone's opportunity and duty to speak up when behaviors or circumstances threaten safety, excellence, and staff wellbeing (34, 38, 39).

Efforts to improve reporting, however, require more than modeling right behaviors, preaching the importance, mandating training, or acquiring user-friendly reporting systems. If leaders are committed to a reporting culture, they must prioritize having a comprehensive plan to reliably address the challenges posed by a small but sometimes influential number of team members who model disrespect in the workplace. Furthermore, leaders must hold each other accountable to address individuals who will not respond to efforts to promote professional accountability, no matter how much revenue Dr. Surgeon generates (26, 27, 29).

Sometimes behavioral issues are buried within peer review or considered private matters beyond the scope of a multidisciplinary case review. The truth is Dr. Surgeon's behavior was anything but private for the team trying to deliver safe care, to Mr. Patient, and likely the team assigned to conduct a case review to understand why an avoidable outcome occurred in Dr. Surgeon's OR. Tepid responses like re-evaluating policies, re-educating staff, and audits for 6 weeks to 6 years do little to protect safety if unprofessional behaviors are not acknowledged, reported, and addressed (30, 31).

Clinicians such as Dr. Surgeon can seem to have immunity from consequences and as a result may not get the wellness help they might need. Some safety experts may rationalize, “Dr. Surgeon surely did not come to work today to cause harm,” as though the absence of overt intent somehow absolves the disrespectful clinician from responsibility for the harm their behavior causes for patients and coworkers alike (41). Is it right to question intent or question awareness? When entering an OR, ICU, or ED, does Dr. Surgeon ever pause and reflect about how other professionals can be supported in doing their work error free? Is Dr. Surgeon capable of recognizing a personal role in creating “chaos” (Dr. Surgeon's description) or a “toxic work environment” (the colleagues' reality) including the harmful impact on Mr. Patient's care and everyone else in the OR? How will Dr. Surgeon gain insight into his professional duty to reflect and self-regulate or seek help unless fellow professionals such as Dr. Sleep or Dr. Leader engage directly? (30, 31, 34). With respect to Dr. Surgeon, there likely was no intent to harm, that said, Mr. Patient still suffered an avoidable complication.

## Fulfilling responsibilities to Mr. Patient

To close the loop, the Leadership Team met several times with Mr. Patient following a concise communication plan:

• Reinforce the apology and convey a future commitment to Mr. Patient's care.
• Correct Mr. Patient's impression that the OR team was incompetent, and inform him that there was more to the story.
• Leadership was committed to addressing the underlying safety issues identified.
• Assign a point person to meet Mr. Patient's care needs arising from the mistake and explore fair compensation for the harm caused (42).

Reasons that compel patients who believe they have been injured to seek help from an attorney are well known and include three that might be important to Mr. Patient: receipt of conflicting information from influential others (often medical professionals who have “different” ideas of what happened); perceptions of a cover-up (no one told me about the staffing problems); and fear that other patients will experience the same outcomes (4–6). Well-run CRPs anticipate and seek to address motivating interests. Despite assurances from Leadership that Mr. Patient would not be treated differently if he consulted a lawyer, he never did. The RFB was removed, his other medical needs were addressed, and Mr. Patient's physical recovery proceeded. Although the philosophical and practical components of patient compensation are beyond the scope of this article, suffice to say that care was spent calculating the financial, future, and emotional implications of the mistake, tangible and intangible, and a fair settlement was offered and accepted.

## A CRP failure?

The Leadership Team recognized gaps in their CRP process, gaps that allowed Dr. Surgeon to spin an embarrassing and inaccurate explanation to a patient harmed directly by his disrespectful OR conduct. The CRP, however, was far from a failure.

In a hospital that followed a traditional, litigation-focused approach, Mr. Patient's case might never have come to the attention of Leadership. The clinical harm was thankfully limited in time and severity. The financial exposure was not catastrophic. The mistake was not “defensible.” To professionals focused entirely on financial exposure, these factors would have likely resulted in a quiet and modest settlement with little more than a line-item buried on a risk management report. Even in an evolving CRP process, leaders heard firsthand from Mr. Patient about the mistake and its consequences. As clinical leaders they recognized the imminent safety threats posed by Dr. Surgeon and the disturbing culture that enabled dangerous, disrespectful behavior hidden by the complicit silence of a considerable clinical audience. The CRP, as challenged as it was, identified hard truths, revealed missed opportunities by leadership to support their culture of safety and address what easily could lead to more serious harm experienced by others. Additionally, the CRP and resulting leadership actions represented steps forward in seeking to demonstrate to the clinical audience that safety should be a real priority, and not just a box to check.

To understand why the CRP initially did not uncover Dr. Surgeon's disrespectful behaviors and why clinical staff members do not report, leadership tasked the Risk Management Director, General Counsel, and Chief Quality and Safety officer to conduct a “root cause analysis” of the CRP. Years of past cases were examined retrospectively for evidence of disrespectful behavior as a contributing factor to avoidable outcomes. There was little evidence of any past plan to address the behaviors and individuals identified. Not surprisingly, Dr. Surgeon was identified in three cases before Mr. Patient's surgery. Earlier recognition and intervention might have spared Mr. Patient and the OR team their traumatic experience. Although seemingly minor to the professional liability community, Mr. Patient's case yielded a rich opportunity to promote patient safety and coworker wellbeing. Leadership regarded a principled response including Mr. Patient's settlement as an investment in their own health system and the system's support of the pursuit of professionalism. The audience of healthcare professionals involved in Mr. Patient's care witnessed their organization serving their mission statement through compassionate and principled treatment of a harmed patient. An even larger audience saw their colleagues' courage recognized for speaking up with overdue attention noticeably paid to addressing disrespect in the workplace and supporting Dr. Surgeon's wellbeing. And Dr. Surgeon is grudgingly grateful for the insight, and professional support received, and the chance to move forward as a professional.

Organizations cannot improve if they do not first identify risks, whether related to dysfunctional systems or challenging humans. Once identified, leaders have a moral and ethical responsibility to understand and address the challenges identified to protect other patients and team members, evaluating the issues with honesty and communicating the lessons learned widely and thoughtfully (43). CRPs will reveal hard-to-hear and sometimes, as in Mr. Patient's case, hard-to-face truths. To gain the full benefit of what CRPs promise, leaders must be prepared to value the lessons learned, be willing to look into the mirror themselves on occasion, and act to support and sustain the pursuit of a culture of safety and respect.

## Data Availability

The original contributions presented in the study are included in the article/Supplementary Material, further inquiries can be directed to the corresponding author.
